# Indirect Calorimetry to Measure Metabolic Rate and Energy Expenditure in Psychiatric Populations: A Systematic Review

**DOI:** 10.3390/nu15071686

**Published:** 2023-03-30

**Authors:** Joshua Daniel Di Vincenzo, Liam O’Brien, Ira Jacobs, Muhammad Youshay Jawad, Felicia Ceban, Shakila Meshkat, Hartej Gill, Aniqa Tabassum, Lee Phan, Sebastian Badulescu, Joshua Daniel Rosenblat, Roger S. McIntyre, Rodrigo B. Mansur

**Affiliations:** 1 Mood Disorders Psychopharmacology Unit, University Health Network, Toronto, ON M5T 2S8, Canada; joshua.divincenzo@uhnresearch.ca (J.D.D.V.);; 2 Faculty of Kinesiology and Physical Education, University of Toronto, Toronto, ON M5S 2W6, Canada; liamt.obrien@mail.utoronto.ca (L.O.); ira.jacobs@utoronto.ca (I.J.); 3 The Tannenbaum Institute for Science in Sport, University of Toronto, Toronto, ON M5S 2W6, Canada; 4 Brain and Cognition Discovery Foundation, Toronto, ON M4W 3W4, Canada; 5 Department of Psychiatry, University of Toronto, 250 College Street, 8th Floor, Toronto, ON M5T 1R8, Canada

**Keywords:** metabolism, depression, bipolar, schizophrenia, respiratory quotient, respiratory exchange ratio, substrate utilization

## Abstract

Psychiatric and metabolic disorders are highly comorbid and the relationship between these disorders is bidirectional. The mechanisms underlying the association between psychiatric and metabolic disorders are presently unclear, which warrants investigation into the dynamics of the interplay between metabolism, substrate utilization, and energy expenditure in psychiatric populations, and how these constructs compare to those in healthy controls. Indirect calorimetry (IC) methods are a reliable, minimally invasive means for assessing metabolic rate and substrate utilization in humans. This review synthesizes the extant literature on the use of IC on resting metabolism in psychiatric populations to investigate the interaction between psychiatric and metabolic functioning. Consistently, resting energy expenditures and/or substrate utilization values were significantly different between psychiatric and healthy populations in the studies contained in this review. Furthermore, resting energy expenditure values were systematically overestimated when derived from predictive equations, compared to when measured by IC, in psychiatric populations. High heterogeneity between study populations (e.g., differing diagnoses and drug regimens) and methodologies (e.g., differing posture, time of day, and fasting status at measurement) impeded the synthesis of results. Standardized IC protocols would benefit this line of research by enabling meta-analyses, revealing trends within and between different psychiatric disorders.

## 1. Introduction

Psychiatric and metabolic disorders constitute two of the greatest burdens to the health of individuals globally. The disability-adjusted life years (DALYs) incurred by psychiatric and metabolic disorders have been continually rising across most of the world [1,2]. Elevated systolic blood pressure, fasting glucose, and body-mass index (BMI) were identified as the 1st, 3rd, and 4th leading risk factors for DALYs, respectively [1,2]. Psychiatric disorders also rank among the top 10 leading causes of global disease burden and show no signs of decreasing incidence [2].

Moreover, mounting evidence indicates a bidirectional, longitudinal relationship between metabolic and psychological wellbeing [3]. Evidence suggests that the years of life lost due to psychiatric disorders are often underestimated, and do not truly reflect the true morbidity and high premature mortality rates observed in populations with psychiatric disorders [1]. Furthermore, greater than half of the excess mortality observed in psychiatric populations is attributable to cardiovascular diseases or other metabolic conditions (e.g., diabetes mellitus, non-alcoholic fatty liver disease, obesity, cholesterol disorders, etc.), which are not adequately treated, and sometimes exacerbated, by currently available psychiatric therapies like some antidepressants and antipsychotics [4,5]. Conversely, preliminary evidence suggests that insulin resistance in the brain contributes to the pathophysiology of psychiatric disorders, and that drugs which increase insulin sensitivity could be effective in treating some psychiatric patients [6]. The foregoing indicates that metabolic comorbidities are overly represented in patients with psychiatric disorders, and that these comorbidities might be more modifiable and higher-priority treatment targets to optimize quality of life and patient well-being [4,5].

For the purposes of this review, metabolism refers to the biochemical processes wherein organisms convert chemical energy from food into the cellular energy and building blocks (i.e., proteins, lipids, carbohydrates, and nucleic acids) required to sustain life. Adenosine triphosphate (ATP) is the primary chemical energy currency in eukaryotic cells. At rest, cellular energy homeostasis is maintained primarily by the oxidation of intracellular carbohydrate, lipid, and protein stores to facilitate the biochemical reactions necessary to synthesize ATP for cellular energy [7]. Oxidation of these substrates yields carbon dioxide (CO_2_), water (H_2_O), and heat as metabolic by-products [8]. Additionally, comparatively small amounts of ATP are produced through anaerobic energy pathways involving anaerobic glycolysis and the phosphagen system [7,9]. The total energy expenditure (TEE)—defined as the total heat energy released by the human body per day [10]—of an individual can be divided into three components: (1) resting energy expenditure (REE), which refers to the energy requirements necessary to establish energy homeostasis at rest; (2) activity energy expenditure (AEE), which refers to the energy requirements necessary to establish energy homeostasis during physical movement; and (3) the thermogenic effect of food, which refers to the energy requirements necessary to digest, absorb, and store nutrients from food.

As a large percentage of REE is derived from oxidative reactions, the associated metabolic rates can be validly and reliably estimated based on the rate of oxygen (O_2_) consumption and CO_2_, H_2_O, and heat production [11]. In laboratory settings, measurement of human energy expenditure (EE) can be achieved via direct or indirect calorimetry (IC) methods.

Direct calorimetry is considered the “gold standard” for assessing EE in humans and involves determining EE within a thermally isolated chamber that precisely measures changes in temperature that can only be attributed to metabolic heat production. Due to the relative unavailability of human direct calorimeters, IC methodologies that are more practical for clinical research purposes have been developed to reliably estimate human EE. The IC technique considered the most valid and reliable for estimating average EE in free-living humans over a period of days is the doubly labelled water method (DLW), which involves the oral ingestion of the stable isotopes deuterium (^2^H) and ^18^O and subsequent measurement of their dilution and elimination rates [12,13,14]. The valid implementation of this technique involves well controlled, complex, and expensive analytical techniques, which make the DLW method one that has not been broadly adopted by researchers [12]. In contrast, respiratory gas exchange systems are a widely used, relatively non-invasive IC methodology for the real-time assessment of human EE.

The respiratory gas exchange method for determining metabolic rate is based on the measurement of inspired and expired respiratory gases for the calculation of the volume and rate of oxygen consumption ($\overset{\cdot }{V}$O_2_) and carbon dioxide production ($\overset{\cdot }{V}$CO_2_). The ratio between $\overset{\cdot }{V}$CO_2_ and $\overset{\cdot }{V}$O_2_, known as the respiratory exchange ratio (RER) in organisms, can be used to quantify the substrate(s) being oxidized to fuel energy metabolism by comparing measured RER values to the known RERs associated with the complete oxidation of different macronutrients [8]. When at a metabolic steady state, RER typically ranges between 0.7 and 1.0, with the former indicating that 100% of oxidative metabolism is fueled by lipids and the latter indicating that 100% of metabolism is fueled by carbohydrates. A steady-state RER of ~0.85 indicates that oxidative metabolism is fueled by a mixture of carbohydrate and fat oxidation with relatively minimal contributions from protein metabolism in healthy, non-fasting individuals [15]. Respiratory gas exchange is well established as a reliable and valid method for calculating EE in resting and exercising animals, including humans, for periods ranging from a few minutes to several hours, and has been widely used with human research participants for nearly a century [16,17,18].

While the complex bidirectional relationship between metabolic and psychiatric health continues to be elucidated, a paucity of studies have directly investigated energy expenditure in psychiatric populations. Notwithstanding, evidence has begun to emerge suggesting metabolic perturbations in populations with psychiatric disorders due to lifestyle (e.g., increased sedentary behaviour in depression), iatrogenic (e.g., antipsychotics or antidepressants associated with increased fat mass and low-density lipoprotein cholesterol), and other unknown factors [19]. Thus, as interest in the metabolic bases of psychiatric conditions grows, it is necessary to synthesize the extant literature on EE testing in psychiatric populations to identify/create best practices and inform the methodologies of future studies. The aim of this systematic review is to summarize the methodologies and observations from studies which have used IC to measure metabolic rate/energy expenditure in adult psychiatric populations.

## 2. Methods

### 2.1. Search Strategy

**Figure 1:**
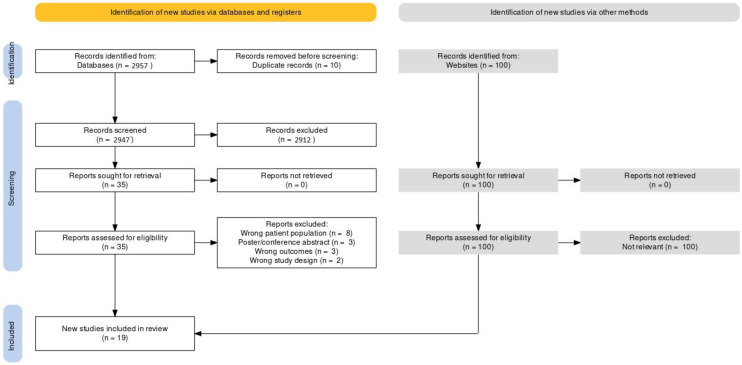
PRISMA flow diagram of search results.

An open search was conducted on the EMBASE, PsycInfo, and PubMed databases, as well as ClinicalTrials.gov and the first 10 pages of Google Scholar, through 26 April 2022, using the following string: (Psychiatr*[MeSH Terms]) AND (indirect calorimet*[MeSH Terms]). Additionally, any relevant references identified in articles from the preliminary search were included to capture any other relevant extant literature. After conducting this search, 95 reports were identified. Upon correspondence with collaborating authors, it was determined that a secondary search was necessary to broaden the capture of relevant articles assessing metabolic rate in psychiatric populations. An additional search was performed on 30 June 2022, on PubMed, using the following search string: (Psychiatr*) AND ((indirect calorimet*[MeSH Terms]) OR (oxygen consumption [MeSH Terms]) OR (VO2 [MeSH Terms]) OR (metabolic rate [MeSH Terms]) OR (Energy expenditure [MeSH Terms]) OR (resting metabolic rate [MeSH Terms]) OR (REE [MeSH Terms]) OR (basic metabolic rate [MeSH Terms]) OR (BMR [MeSH Terms])). This search identified 2862 additional records that were incorporated into the review. In total, 14 of the reports included in the current review were identified in the first search, while 5 more relevant reports were included from the second search. The raw search results were exported to covidence.org and two reviewers (JD and LO) performed the title and abstract review, full-text review, and data extraction, with discrepancies resolved by discussion with input from a third party (RM) until consensus was reached. A PRISMA flow diagram (Figure 1) was used to organize the search results and Mendeley Desktop 1.19.8 was used to manage references and deduplication. Due to the high heterogeneity and small number of available studies (mainly consisting of non-randomized or observational/cross-sectional studies), statistical synthesis was not considered viable.

### 2.2. Study Selection and Eligibility Criteria

Original interventional and observational studies including clinical trials, retrospective studies, and open-label studies were eligible for inclusion in the first search if they were conducted in adult humans and published in English. Studies that used IC to assess resting metabolic rate in populations with psychiatric disorders were included in the current review. Review papers, conference, and poster presentations were excluded to avoid capturing the same study sample twice. We also excluded studies in which methods other than IC were used to assess metabolic rate (e.g., equation-derived estimation using anthropometrics), studies involving participants with eating disorders or neurodegenerative diseases (to avoid confounding effects on metabolism due to closely linked pathogenesis), and studies involving individuals <18 years of age.

### 2.3. Quality Appraisal Strategy

Risk of bias was measured using the Newcastle–Ottawa Scale for non-randomized case-control and cohort studies [20], the ROBINS-I tool for non-randomized interventional studies [21], the ROB2 tool for randomized trials [22], and the Joanna Briggs Institute (JBI) critical appraisal checklist for analytical cross-sectional studies [23].

## 3. Results

### 3.1. Search Results

After deduplication of 2957 studies identified in the search, 2947 studies remained. The titles and abstracts of these 2947 studies were screened and 2912 irrelevant studies were excluded, leaving 35 studies for which the full texts were assessed for eligibility. Of the remaining 35 studies, 16 were excluded, and thus, the final synthesis and review included 19 studies (Figure 1) [24,25,26,27,28,29,30,31,32,33,34,35,36,37,38,39,40,41,42].

### 3.2. Quality Appraisal Results

Results of the quality appraisal are shown in Table 1. Overall, the studies by Caliyurt et al. [24], Hassapidou et al. [30], Gaist et al. [27], and Virkkunen et al. [41] were deemed to have a moderate risk of bias due to confounding factors for which reasonable attempts were made by the authors to mitigate their impacts, whereas Gewirtz et al. [29] was deemed to have a serious risk of bias due to confounding factors with no attempt to assess and/or mitigate their potential impacts. The rest of the studies were deemed to be of high methodological quality.

### 3.3. Characteristics and Findings of Included Studies

Of the 19 studies included, 8 were cross-sectional studies, 6 were case-control studies, 4 were non-randomized interventional studies, and 1 was a randomized controlled trial. There were 6 studies conducted in the United States, 3 in Australia, 2 each in Italy, Greece, and Finland, and 1 each in Japan, South Korea, Sweden, and Turkey. The sample sizes ranged from 8 to 989 participants. In aggregate, 1875 participants were enrolled across the 19 included studies as follows: 198 healthy controls (HCs); 92 bipolar-I disorder (BD-I); 287 schizophrenia/schizophreniform disorder; 10 seasonal affective disorder (SAD); 15 treatment-resistant depression (TRD); 1117 severe mental illness (SMI); 11 major depressive disorder (MDD); 145 habitually violent offenders with antisocial personality disorder (APD). Of the 19 included studies, 18 used only respiratory gas exchange measurements as the method of indirect calorimetry and metabolic rate calculations; whereas one study used a respiratory gas exchange method to measure REE and used the DLW method to measure TEE over 10 days. Moreover, 15 studies were observational, while 4 were interventional, investigating the effects of a prescribed Mediterranean diet, bright light therapy, or various drugs on body composition and metabolism.

There was a notable degree of heterogeneity in the IC protocols reported by each study with respect to the sampling duration, time of day, digestive state, and postural positioning, as well as how each study defined “steady state” and the participants being “at rest”, all of which may affect IC measurements. While in summary the results herein indicate that REE is consistently altered in psychiatric patients compared to healthy controls, the directionality (i.e., whether/when REE is increased or decreased) and whether these alterations represent a cause or rather a consequence of the psychiatric condition, remain to be illuminated. The IC methodologies and results of each study are described in Table 2.

#### 3.3.1. Resting Energy Expenditure in Psychiatric versus Control Participants

In studies where absolute REE values were similar between psychiatric patients and controls, differences in substrate utilization could be observed as patients tended to exhibit reduced lipid oxidation compared to healthy controls [26,37]. Some studies reported higher REE values in patients with Bipolar I Disorder [24] and Seasonal Affective Disorder in off-light conditions during the winter [27], whereas other reports found consistently lower REE and/or non-oxidative glucose metabolism in psychiatric patients [33,41,42]. One study found no difference in measured REE between BD-I patients and healthy controls [39], and another study found that schizophrenia patients had higher RER than controls, but REE did not differ between groups when corrected for FFM [37].

#### 3.3.2. Predictive Equations versus Indirect Calorimetry to Measure Energy Expenditure

Predictive equations for metabolic rate/EE were not as accurate in psychiatric patients as they were in healthy controls [32,36,38,39,40]. Four studies demonstrated commonly used predictive REE equations to systematically overestimate REE in psychiatric patients [36,38,39,40]. Between studies, there were discordant results in the suitability of specific predictive equations at estimating REE. For example, three studies found the Harris–Benedict equation to overestimate REE [36,38,39], whereas Sugawara et al. [40] did not find significant predictive bias in the Harris–Benedict equation in patients with schizophrenia or schizoaffective disorders. Furthermore, Miniati et al. [32] found that REE values measured by IC differed significantly from those estimated using three commonly used predictive equations, thus rendering the equations clinically inappropriate for estimating REE in female patients with BD-I, compared to IC.

## 4. Discussion

The present systematic review sought to summarize the methodological and observational data made available in the English literature reporting metabolic rate and/or energy expenditure data measured by IC methodologies in human psychiatric populations. In general, the studies included herein reported mixed findings, mainly due to the high degree of heterogeneity between study objectives and methodologies. As measured EE values are highly dependent on the IC protocol employed (i.e., time spent resting, body positioning, time of day, digestive status), consequently, inter-study comparability is limited. Nevertheless, in the available case-control studies, most investigations observed healthy controls and psychiatric patients to have differences in metabolism, reflected by the measures of REE, TEE, or RER [24,33,37,41,42].

Such disparate between-study results could be due to differences in many factors such as measurement methodologies, pharmacologically-induced alterations in metabolism, or metabolic profiles among different psychiatric illnesses. However, it is noted that the limited evidence does suggest disparate metabolic profiles between those with or without psychiatric illness, and potentially between those with different psychiatric illnesses (Table 2). Characterizations of these metabolic differences, and whether their relationship to the psychiatric illness are correlative or causative, are still to be determined. Moreover, the duration/chronicity and severity of psychiatric disease could affect REE values directly or indirectly through changes in food intake, intake, eating behaviour, and the accompanying changes in body composition. For example, reduced appetite and low energy are common symptoms of depressive episodes [43]. Over time, reduced caloric intake coupled with low levels of physical activity can cause changes in body composition and reductions in REE. The stability of the disease state in this case could mediate the change in REE, as a more chronic depressive phenotype would confer greater metabolic aberrancies relative to healthy controls or individuals with less chronic depressive phenotypes.

Another general finding from this review was that the correlations between measured and predicted EE values were largely weak, especially in patients. This is perhaps due to underlying metabolic aberrations or medications often present in the patient populations [3,44]. Nevertheless, this highlights the importance of diligent IC methodologies and the use of gas exchange IC devices when feasible, rather than predictive equations, for determining EE in psychiatric populations. Taken together, this evidence suggests that the commonly used predictive REE equations are likely unreliable and invalid for estimating REE in psychiatric patients, regardless of whether they are accurate for healthy controls. Further research efforts are necessary to develop valid and reliable predictive equations for use in psychiatric populations.

Drug therapy can aggravate or alleviate metabolic aberrations that may be present in psychiatric patients. Many antipsychotic drugs act by influencing monoaminergic neurotransmission in distinct brain regions known to influence eating behaviour. For example, olanzapine and clozapine have high affinity for the 5-HT2C receptor, which serve important functions in the regulation of motor behaviour and appetite [45]. As such, consumption of these antipsychotics often precipitates increases in food intake and body weight, whereas other antipsychotics such as aripiprazole are less problematic from a weight-gain perspective, due in part to a lower affinity for 5-HT2C receptors [46]. Heterogenous methodologies and patient populations, as well as the small sample sizes of the studies included in this review, precluded direct comparisons of the effects of different drug classes on metabolic responses. Randomized, controlled studies comparing multiple classes of drugs within a homogenous patient population, as well as studies comparing the same drugs in different patient populations, will help elucidate these effects. A preponderance of studies focused on investigating the effects of antipsychotics or other psychiatric drugs on EE, without a control group of medication-free psychiatric participants. Although clinically relevant and representative of naturalistic samples, this leaves a knowledge gap in the overall characterization of EE in psychiatric patients. Pharmacological intervention involving antipsychotic or mood-modulating drugs may also have indirect effects on EE by changing voluntary movement patterns due to altered motivational states. This may lead to systematic over- or under-estimation of EE in psychiatric populations that is a function of the drug, rather than the illness per se. For example, ref. [35] assessed TEE and AEE in male schizophrenia patients undergoing pharmacological intervention with clozapine for symptom management. The authors found that TEE was significantly lower than World Health Organization recommendations, due largely to sedentarism in people with schizophrenia who take clozapine. It is unclear what to make of these results, however, as there were no direct comparisons made to healthy age-matched controls or patients not taking clozapine. Additionally, the use of pharmacological interventions in the available research makes it difficult to quantify if differences in metabolic activity in psychiatric patients are attributed to psychiatric illness or a consequence of the drug response. Without further dedicated efforts to classify the unique metabolic profiles of psychiatric patients, it is difficult to elucidate the effects of psychiatric illness on metabolism.

Overall, 18 studies with low or moderate risk of bias fit the inclusion criteria for review, while one included study had a high risk of bias. Due to the paucity of available studies and high heterogeneity among investigations, no meta-analysis could be conducted as valid mathematical analyses were not viable in this review. Despite this limitation, the trends observed and discussed herein merit reporting and future consideration. This systematic review reinforces the importance of methodological consistency and rigor for obtaining valid and reliable measurements of EE via IC in psychiatric patients to enable comparisons between subjects as well as between groups. For example, it is important that patients are conclusively in a rested and relaxed state, as even slight fidgeting or anxiousness during IC measurements can influence REE outcomes and can often go unrecognized by the examiner. For this reason, it is recommended that IC measurements should last at least 20–30 min to allow sufficient time for individuals to reach a true resting equilibrium. All included studies in this review which reported the timing of IC assessments measured participants for at least 20 min, however some groups [32,41,42] did not provide details on the timing of the IC assessments. Thus, methodological variability can increase uncertainty and introduce undue heterogeneity in the interpretation and translation of the results to a broader synthesis of evidence. Best practices for respiratory gas exchange IC have not yet been established or agreed upon by the scientific community, so researchers must develop and validate their own protocols, which contributes to the high methodological heterogeneity. The creation of a consortium of scientists, clinicians, and researchers with the goal of establishing an international, gold-standard methodology for respiratory gas exchange IC would be an important step toward reducing the issue of methodological heterogeneity.

## 5. Conclusions

At present, a scarcity of research has investigated IC in psychiatric populations. Based on the available evidence, it appears that the metabolic profiles of psychiatric patients may be discordant to those of healthy controls. Furthermore, the results herein indicate that predictive equations used to estimate EE in healthy individuals are likely inadequate for estimating EE in psychiatric patients for reasons that remain unclear, warranting further investigation. Lastly, studies examining the effects of pharmacological intervention on metabolic rate confound the interpretation of the unique effects of psychiatric illness on bioenergetics. Further research efforts are necessary to elucidate the complex relationships among energy metabolism, the brain, and behaviour, thus enabling future meta-analyses.

## Figures and Tables

**Table 1:** Results of quality appraisal.

**Newcastle** **–Ottawa Quality Assessment Scale for Case-Control Studies**									
**Author (Year)**	**Selection**			**Comparability**			**Exposure**		
Caliyurt (2009) [24]	4/4			2/2			0/3		
Nilsson (2006) [33]	4/4			2/2			2/3		
Virkkunen (2007) [42]	4/4			2/2			2/3		
Soreca (2007) [39]	4/4			2/2			2/3		
Fleet-Michaliszyn (2008) [26]	3/4			2/2			2/3		
Sharpe (2009) [37]	4/4			2/2			2/3		
**Risk of Bias in Non-Randomized Studies—Interventional**									
**Author (Year)**	**Confounding Bias**	**Selection Bias**	**Classification Bias**	**Deviation Bias**	**Missing Data Bias**	**Measurement Bias**	**Reporting Bias**		**Overall Bias**
Hassapidou (2011) [30]	Moderate	Low	Low	Low	Low	Low	Moderate		Moderate
Virkkunen (2009) [41]	Moderate	Low	Low	Low	Low	Low	Low		Moderate
Gaist (1990) [27]	Moderate	Low	Low	Low	Low	Moderate	Low		Moderate
Gewirtz (1988) [29]	Serious	Low	Low	Low	Low	Moderate	Low		Serious
**Joanna Briggs Institute Critical Appraisal Checklist for Analytical Cross-Sectional Studies**									
**Author (Year)**	**Domain 1**	**Domain 2**	**Domain 3**	**Domain 4**	**Domain 5**	**Domain 6**	**Domain 7**	**Domain 8**	**Overall Appraisal**
Fan (2006) [25]	Yes	Yes	Yes	Yes	Yes	Yes	Yes	Yes	Include
Graham (2005) [28]	Yes	Yes	Yes	Yes	Yes	Yes	Yes	Yes	Include
Miniati (2015) [32]	Yes	Yes	Yes	Yes	Yes	Yes	Yes	Yes	Include
Sharpe (2005) [36]	Yes	Yes	Yes	Yes	Yes	Yes	Yes	Yes	Include
Sharpe (2006) [35]	Yes	Yes	Yes	Yes	Yes	Yes	Yes	Yes	Include
Skouroliakou (2009) [38]	Yes	Yes	Yes	Yes	Yes	Yes	Yes	Yes	Include
Sugawara (2014) [40]	Yes	Yes	Yes	Yes	Yes	No	Yes	Yes	Include
Herman (1991) [31]	Yes	Yes	Yes	Yes	Yes	No	Yes	Yes	Include
**Revised Cochrane Risk-of-Bias Tool for Randomized Trials (RoB 2)**									
**Author (year)**	**Randomization Process**	**Deviations from Intended Interventions**		**Missing Outcome Data**	**Measurement of the Outcome**		**Selection of the Reported Result**		**Overall Bias**
Park (2013) [34]	Low	Low		Low	Low		Low		Low

**Table 2:** Results of systematic review and synthesis.

Author (year)	Country	Type of Study/ Design	*N*	Mean Age in Years (SD)	Population	Intervention (Formulation, Dose, Regimen)	Concomitant Medications (*N*)	Neuropsychiatric Assessments	Calorimetry Method Used	Main Outcomes
Caliyurt (2009) [24]	Turkey	Observational/case control	69 (42 BD-I; 27 HC)	36.45 (11.23)	BD-I and HCs	No intervention	BD group only: Lithium (24), Chlorpromazine (17), Olanzapine (16), Valproic acid (13), Clonazepam (4), Quetiapine (4), Risperidone (3), Clozapine (1), Carbamazepine (1), Sulpiride (1), Oxcarbazepine (1)	BRMAS	REE was measured via gas exchange IC in the fasted state. Subjects rested in the supine position for 30 min before a 20 min measurement was taken. ‘Steady state’ was determined by device software as 5 min where average $\overset{\cdot }{V}$O_2_ changed by less than 10% and RER changed by less than 5%. REE was determined based on an abbreviated Weir equation.	REE was higher in the BD-I group vs. controls. Controls showed significant correlations between BMI and REE that were not replicated in the BD-I group. There was also no relation between BRMAS scores and REE values in BD-I patients.
Fan (2006) [25]	United States	Observational/cross sectional	71	42.2 (10.5)	Outpatients with schizophrenia or schizoaffective disorder, without diabetes or other significant medical illnesses	No intervention	Clozapine (24), olanzapine (22), risperidone (16), quetiapine (4), ziprasidone (2), medication free (3)	None	REE was measured via gas exchange IC in the fasted state. Subjects were rested with a canopy placed over their head for the collection of expiratory gases. REE was calculated using a standard equation involving the measured RER.	REE measured using the metabolic cart significantly correlated with REE predictions derived from all four equations that were analyzed (Harris–Benedict adjusted and current body weights, Schofield, and Mifflin–St. Jeor) (*p* < 0.001). The Mifflin–St. Jeor equation correlated most strongly with the metabolic cart measurements, whereas the Harris–Benedict current body weight and Schofield equations significantly overestimated REE (*p* < 0.001) in SMI patients taking olanzapine.
Fleet-Michaliszyn (2008) [26]	United States	Observational/case control	35 (18 BD-I; 17 HCs)	BD-I = 41.4 (2.1); HCs = 40.9 (2.3)	Women with BD-I, euthymic and treated with ≤3 medications, who never received olanzapine and/or clozapine; and HCs	No intervention	Antipsychotic medications (12; aripiprazole, ziprasidone, haloperidol, quetiapine, or perphenazine), lithium and anticonvulsant medications (17; valproate, carbamazepine, lamotrigine, gabapentin, or topiramate), SSRIs (5; escitalopram, fluoxetine, or sertraline), SNRI (2; venlafaxine), and benzodiazepines (3; lorazepam or clonazepam). Two control subjects (1 obese and 1 normal weight) were treated with antihypertensive agents.	None	REE and substrate oxidation were measured via gas exchange IC in the fasted state. Subjects were rested with an open canopy system placed over their head for the collection of respiratory gases. Measurements were collected for 35 min with the first 5 min of data discarded. Data were obtained at 30 s intervals. Substrate oxidation was determined using a standard equation and TEE and AEE were estimated using an armband that was worn for 5 days.	BD-I patients and healthy controls had comparable insulin resistances (mean ± SEM HOMA-IR = 2.7 ± 0.7 vs. 2.5 ± 0.7, for patients and controls, respectively; *p* = 0.79). BD-I patients had 13% lower fat oxidation at rest than HCs, but resting metabolic rates were comparable. Activity monitors revealed that neither mean total daily energy expenditure, nor energy expenditure during PA was different between patients and controls. There were also no differences in mean time spent in sedentary, moderate, or vigorous activity.
Gaist (1990) [27]	United States	Interventional/Non-randomized experimental study	19 (10 SAD, 9 HCs)	SAD = 39.1 (5.8); HCs = 38.9 (8.9)	Patients meeting the lifetime criteria for seasonal affective disorder who were drug free for at least a month and scored at least 14 on the 21-item Hamilton Depression Rating Scale. Normal controls were recruited through an advertisement in the Washington Post who were screened and evaluated via a telephone questionnaire, clinical interview, physical examination, and routine laboratory tests.	Bright light exposure therapy	Drug-free for at least 1 month	HAMD	REE was measured via gas-exchange IC after an overnight fast. Patients rested for 2 h with lights either dimmed in the off-light condition or on in the on-light condition. IC measurements were conducted in recumbent position and expired gases were collected for 20 min on a breath-by-breath basis. Data were averaged over 60 s intervals and EE was determined by the Weir equation. REE values were expressed using the DuBois formula to enable comparisons based on body size.	Note: the study was conducted between December and March. SAD patients had significantly higher REE in off-light conditions compared to HCs (mean ± SD = 35.7 ± 3.7 and 30.6 ± 4.9 kcal/m^2^/h, respectively; *p* < 0.02, df = 17); After light treatment, REE was significantly reduced in SAD patients (35.7 ± 3.7 versus 32.4 ± 6.3 kcal/m^2^/h; *p* < 0.05, df = 9), but not HCs (30.6 ± 4.9 and 29.9 ± 3.12 kcal/m^2^/h off-light and on-light treatment, respectively). Also, patients’ mood improved in the on-light condition, as indicated by the HAMD (19.5 ± 5.4 versus 5.4 ± 3.3; *p* < 0.001).
Gewirtz (1988) [29]	United States	Interventional/Case series	15	Range = 24–59	Women aged 24–59 with depression refractory to outpatient medication	Thyroxine, liothyronine; various doses; daily	Tranylcypromine (Patient 1 only)	Not reported	Metabolic rate was assessed using gas-exchange IC. Subjects were fasted with a canopy placed over their head for the collection of respiratory gases. Patients were monitored for 30 min and measurements were taken once a steady state was achieved. Heat production per day was inferred based on O_2_ consumption and CO_2_ production data.	There was 6 out of the 15 patients with evidence of occult hypothyroidism, all of whom responded to thyroid hormone medication and achieved a normal metabolic rate and/or thyroid hormones, with a reduction in depression, except for one who was lost to follow up. The remaining 9 participants were euthyroid, and/or eumetabolic or hypermetabolic and thus thyroid hormone interventions were not initiated.
Graham (2005) [28]	United States	Cohort study	9	median age = 21.5 years (range = 20.8–27.3)	Adults with DSM-IV diagnosis of brief psychotic disorder, schizophreniform disorder, schizophrenia, or schizoaffective disorder, without other active illnesses and with no history of antipsychotic drug therapy	Olanzapine titrated by the treating physician in 2.5 mg increments, with final doses ranging between 2.5–20 mg/day based on clinical response.	Not reported, but the following medications were allowed: lorazepam, clonazepam, zolpidem, benztropine, and propranolol	None	RER and REE were determined via gas exchange IC 2 h postprandial. After resting supine for 30 min, $\overset{\cdot }{V}$O_2_ and $\overset{\cdot }{V}$CO_2_ were measured at 30 s intervals for 20 min. REE was determined using the Weir equation.	Median increase in body weight was 4.7 kg after ~12 weeks, a significant increase of 7.3% from first observation. Body fat, measured by dual-energy X-ray absorptiometry, increased significantly, with a propensity for central fat deposition. Lean body mass and bone mineral content did not change. Absolute 24 h REE and REE normalized to lean body mass did not change with 12 weeks of olanzapine treatment. Baseline resting energy expenditure was not lower in subjects who gained weight. RER increased significantly with olanzapine treatment (~14%) and was positively correlated with change in weight (r = 0.73). Fasting insulin, C-peptide, and triglyceride levels significantly increased, but there were no changes in glucose levels; total, high-density lipoprotein, or low-density lipoprotein cholesterol levels; or leptin levels. Results suggest decreased fatty acid oxidation and a shift toward carbohydrate oxidation, with possible development of insulin resistance.
Hassapidou (2011) [30]	Greece	Interventional/Non-randomized experimental study	Enrolled *n* = 989; Completed *n* = 145	40 (11.7)	Psychiatric patients with severe mental illness. At baseline, all patients were classified as obese (mean bodyweight = 94.9 ± 21.7 kg; mean BMI of 34.3 ± 6.9 kg/m^2^).	A dietary regimen designed to produce a weekly caloric deficit of 500 kcal, characterized by moderate consumption of carbohydrates (50–55% of total energy per day) and a high fiber content, 15–20% protein and a fat intake of 30–35% of total energy per day. Patients were advised to consume fruits, vegetables, and whole grains daily and to increase their consumption of olive oil.	Maintained stable regimens throughout the study period: antidepressants (297, 30%); antipsychotics (274, 28%); antipsychotics + antidepressants (230, 23%), antipsychotics + antidepressants + other (188, 19%)	None	REE was measured via gas exchange IC. Subjects completed an activity questionnaire and an activity factor of 1.3 to 1.5 was multiplied to REE to determine energy requirements. Description of methodology (i.e., preparations and sampling duration) for IC protocol is absent from manuscript.	Progressive statistically significant reductions in mean weight, fat mass, waist circumference, and BMI throughout the duration of monitoring (*p* < 0.001). The mean final weight loss was 9.7 kg and BMI decreased to 30.7 kg/m^2^ (*p* < 0.001). The mean final fat mass loss was 8.0 kg, and the mean final waist circumference reduction was 10.3 cm (*p* < 0.001) compared to baseline. Significant and continual reductions were observed in fasting plasma glucose, total cholesterol, and triglycerides concentrations throughout the study (*p* < 0.001). REE decreased significantly in completers at months 3 and 6 compared to baseline (*p* < 0.001).
Herman (1991) [31]	United States	Observational/cross sectional	11 (6 Female; 5 Male)	36 (10.7)	Patients diagnosed with DSM-III major affective disorder, medication free and on a low monoamine diet.	Carbamazepine; 200–1400 mg; daily	Medication free for at least 2 weeks	Bunney–Hamburg Global Rating Scale, HAMD	REE was measured via gas exchange IC using a breath-by breath metabolic cart system. Data were acquired over 20 min with the first 5 min discarded. REE was calculated at 1 min intervals using measured $\overset{\cdot }{V}$O_2_ and $\overset{\cdot }{V}$CO_2_ and a standardized equation. Predicted REE was calculated using the Harris–Benedict equation.	The mean change in REE for all patients was −0.84 kcal/m^2^/h, a 2.7% decrease which was not significantly different. The analysis was also performed separately for the two groups of patients with and without multiple measurements of REE. There were no significant differences between REE before and during CBZ treatment when the two groups of patients were considered separately. Mean baseline REEs were not correlated with the Bunney–Hamburg rating the day of REE measurement, the average of 7 days prior, or the baseline Hamilton rating. The change in depression, measured by the Bunney–Hamburg or Hamilton rating, was also not correlated with the change in REE on CBZ. CBZ blood levels, dosages, or duration of CBZ treatment were not correlated with the change in REE. Low baseline REEs were significantly associated with high exposure to TCA/MAOIs, as well as being a woman. Baseline REEs were significantly correlated with the amount of time the patient was on TCA/MAOIs 5 years prior to admission. There was no relationship between the amount of time a patient was off TCA/MAOIs and the baseline REE. There was no correlation between baseline REEs and the number of months depressed 5 years prior to admission. After carbamazepine treatment, T4 decreased significantly (7.53 versus 5.74 mc/dL, *p* < 0.001), whereas REE did not (31.6 versus 30.7 kcal/m^2^/h). Body weight significantly and positively correlated with baseline REE in men but not women.
Miniati (2015) [32]	Italy	Observational/cross sectional	17	37.3 (11.4)	Female outpatients with BD-I as per the DSM-IV. One patient also met the criteria for obsessive compulsive disorder.	No intervention	Atypical antipsychotics: olanzapine (11; dose range 2.5–15 mg/day), quetiapine (3; dose range 25–100 mg/day), aripiprazole (3, dose range 2.5–15 mg/day). Mood stabilizers: lithium (7; dose range 300–900 mg/day), valproate (3; dose range 500–1200 mg/day), oxcarbazepine (1; 300 mg/day), carbamazepine (1; 300 mg/day), pregabalin (1; 225 mg/day).	None	REE was measured via gas exchange IC in the fasted state. Patients were seated during measurement. Description of sampling duration for IC is absent from the manuscript. Measured REE was compared with predictive REE regression equations: Harris–Benedict, LARN, and Mifflin–St. Jeor.	There was a significant positive relationship between REE and fasting serum insulin level (r = 0.39, *p* = 0.001). Higher fat-free mass and higher fasting serum insulin level predicted increased REE, which could mitigate further weight gain in nondiabetic individuals with schizophrenia.
Nilsson (2006) [33]	Sweden	Observational/case control	47 (30 SCZ; 17 HC)	SCZ = 33.0 (8.7); HCs = 32.3 (7.9)	Physically healthy patients who fulfilled DSM-IV criteria for schizophrenia or schizophreniform disorder, and age- and gender-matched controls with no personal or familiar history of psychiatric disorder.	No intervention	Clozapine (9), olanzapine (5), risperidone (3), haloperidol (1), zuclopenthixol (1); non-medicated (11)	PANSS, GAF	Gas exchange IC was performed in the fasted state using an open ventilated metabolic cart device for measurement of $\overset{\cdot }{V}$O_2_ and $\overset{\cdot }{V}$CO_2_. Participants were resting for a measurement time of 45 min. Gas exchange was collected for 60 s intervals and researchers used last 15 min as resting value. REE was determined according to Weir formula and RER determined from IC device.	REE was significantly lower in the patients than in the controls. A decrease was also seen in the non-medicated patients. The patients showed significantly lower percentages of water in FFM and intracellular water
Park (2013) [34]	South Korea	Interventional/RCT	20 (10 ziprasidone, 10 olanzapine; 50% females in each group)	Ziprasidone = 34.50 (interquartile range [IQR] = 26.25–40.25); Olanzapine = 31.50 (IQR = 26.50–41.25); [*p* > 0.99]	Adults diagnosed with a brief psychotic disorder, schizophrenia, or related disorders according to the DSM-IV and no other active illnesses, free of antipsychotics for at least 3 months. Anxiolytics were permitted.	Ziprasidone or Olanzapine, started at 40 and 10 mg/day, respectively; mean daily doses during the 12-week study period were 109 (range: 65–140) mg/day for ziprasidone and 11.6 (range: 8.2–15.5) mg/day for olanzapine.	Lorazepam, clonazepam, zolpidem, benztropine, or propranolol were the only drugs permitted	DSM-IV Korean Version, PANSS	RER and REE were determined via gas-exchange IC. $\overset{\cdot }{V}$O_2_ and $\overset{\cdot }{V}$CO_2_ were measured at 30 s intervals over 20 min and REE was calculated using a standardized equation.	After 12 weeks of treatment, the percent changes in body weight (*p* = 0.016), BMI (*p* = 0.019), and waist-to-hip ratio (*p* = 0.004) were significantly greater in patients treated with olanzapine than with ziprasidone. REE and RER were not affected by olanzapine therapy, while ziprasidone significantly increased REE normalized to lean body mass; however, between-groups differences in the percent or absolute changes in EE measures were not significant.
Sharpe (2009) [37]	Australia	Observational/case control	62 (31 SCZ, 31 HCs)	SCZ = 34.2 (10.1); HCs = 34.6 (10.1)	Men diagnosed with schizophrenia per the DSM-IV on atypical antipsychotics for at least 4 months; and HCs matched for age and weight adjusted for height. Participants having medical conditions with the potential to affect REE or body composition were excluded.	No intervention	All participants with schizophrenia had been taking an atypical antipsychotic medication [clozapine (15), olanzapine (6), risperidone (7), quetiapine (1), aripiprazole (2)] for more than 4 months as their primary antipsychotic medication	PANSS	REE was measured via gas-exchange IC using a ventilated hood system. Subjects were assessed in the rested state after an overnight fast in the supine position. REE was measured after a 10 min adaptation period and was continuously monitored for 30 min. A total of 10 min of steady state data were acquired and REE was calculated using the abbreviated Weir equation.	The SCZ group showed a significantly lower mean REE than HCs (t = −2.08, *p* = 0.046; 95% CI of difference −178 kcal/day to −2 kcal/day and t = 2.91, *p* = 0.007; 95% CI of difference 0.02 to 0.09). However, after adjusting for FFM, no significant difference between SCZ and HC groups was observed in REE (F = 0.69, *p* = 0.41; 95% CI of difference—124 to +52 kcal/day). Fasting RER was significantly higher in the SCZ group compared to HCs after adjusting for FFM (F = 6.12, *p* = 0.004).
Sharpe (2006) [35]	Australia	Observational/cross sectional	8	28.0 (6.7)	Men with schizophrenia prescribed clozapine for at least 6 months	Clozapine (mean daily dose = 456 ± 143 mg; mean duration = 20.5 ± 12.8 months)	Citalopram (2), amisulpride (1), chlorpromazine (1), diazepam (1), paroxetine (1), risperidone (1), sodium valproate (1) and venlafaxine (1)	None	Free-living TEE was measured via DLW method using oral ingestion of 0.05 g kg^−1^ [^2^H_2_O] and 0.15 g kg^−1^ [H2^18^O]. Urine samples were collected over 10 days. TEE was calculated using multipoint slope–intercept method, with tracer dilution spaces calculated via back extrapolation. REE was measured using gas exchange IC in fasted state. Participants rested for 10 min adaptation before $\overset{\cdot }{V}$O_2_ and $\overset{\cdot }{V}$CO_2_ were analyzed for 30 min. Steady state defined as 10 min where coefficient of variation of $\overset{\cdot }{V}$O_2_ and $\overset{\cdot }{V}$CO_2_ was less than 10%.	The TEE was 2511 ± 606 kcal per day which was significantly lower (by 21%) than WHO recommendations. Physical activity levels confirmed the sedentary nature of people with schizophrenia who take clozapine.
Sharpe (2005) [36]	Australia	Observational/cross sectional	8	28.0 (6.7)	Men diagnosed with chronic paranoid schizophrenia taking clozapine for >6 months, without medical conditions known to affect REE	Clozapine: Dose = 456 ± 143 mg/day; mean months taking clozapine 20.5 ± 12.8	Not reported	None	REE was measured via gas exchange IC using a ventilated hood system. Participants were tested immediately upon waking up, in the fasted state. ‘Steady state’ was defined as 10 min where the coefficient of variation in $\overset{\cdot }{V}$O_2_ and $\overset{\cdot }{V}$CO_2_ was less than 10%.	Harris–Benedict and Schofield equations overestimated resting energy expenditure by ~280 kcal/day for patients taking clozapine. Predictions from the other predictive equations were highly variable and deemed not clinically appropriate for REE estimation.
Skouroliakou (2008) [38]	Greece	Observational/cross sectional	128	41.19 (11.22)	DSM-IV mood or psychotic disorder patients	No intervention	Olanzapine (all subjects; stable for at least 6 months)	None	REE measurement was performed via gas exchange IC in the fasted state. Participants were in the supine position after resting for 10 min. Measurements were conducted for 20 min with the first 5 min of data discarded. ‘Steady state’ data were defined as the 15 min at which the coefficient of variation in $\overset{\cdot }{V}$O_2_ and $\overset{\cdot }{V}$CO_2_ was less than 10%.	REE measured using the metabolic cart significantly correlated with REE predictions derived from all four equations that were analyzed (Harris–Benedict adjusted and current body weights, Schofield, and Mifflin–St. Jeor) (*p* < 0.001). The Mifflin–St. Jeor equation correlated most strongly with the metabolic cart measurements, whereas the Harris–Benedict current body weight and Schofield equations significantly overestimated REE (*p* < 0.001) in SMI patients taking olanzapine.
Soreca (2007) [39]	Italy	Observational/case control	32 (15 BD-I; 17 HCs)	BD-I = 37.13 (range 21–51 years); HCs = 35.59 (range 21–53 years)	Patients with BD-I recruited from the outpatient and day hospital care services at the University of Pisa, in a maintenance treatment phase, taking olanzapine for >6 months. Patients were free from medical comorbidities known to affect REE and weight. HCs were matched for age and gender, with no personal history of mood disorder, and were not on any medication.	The BD-I group was receiving olanzapine, mean dose = 6 mg/day (range 2.5–15 mg/day)	Lithium 600–900 mg/day (6), valproate 500–1000 mg/day (6), paroxetine 20 mg/day (3), sertraline 50–100 mg/day (3), fluoxetine 20 mg/day (1), fluvoxamine 150 mg/day (1), citalopram 20 mg/day (1), venlafaxine 75 mg/day (1)	None	REE was measured via gas exchange IC in the fasted state. Participants rested for 30 min before breathing through a standard mouthpiece for ~10 min of data collection. Device measured $\overset{\cdot }{V}$O_2_ only and assumed an RER of 0.85. Measured REE was compared to predictive REE equations: Harris–Benedict (HB), Schofield (S), LARN, and OUR.	Independent samples *t*-tests showed no significant difference between patients and controls for age and mean measured REE, but mean BMI was significantly greater in the patient group. Paired *t*-tests showed significant differences between expected REE (HB), REE (S), REE (LARN), REE (OUR), and REE measured with IC in the bipolar group for all the equations, with mean expected REE higher than measured REE. Expected REE (HB), (S), (LARN), (OUR), and measured REE did not differ significantly in the control group. The aforementioned equations systematically overestimated REE in BD-I subjects maintained on olanzapine.
Sugawara (2014) [40]	Japan	Observational/cross sectional	110	45.9 (13.2)	DSM-IV schizophrenia or schizoaffective disorder	No intervention	Antipsychotic combination therapy (73), risperidone (13), aripiprazole (11), olanzapine (6), quetiapine (4), blonanserin (2), perospirone (1)	None	REE was measured via gas exchange IC in the fasted state. Participants rested for 30 min before breathing through a standard mouthpiece for ~10 min of data collection.	Measured and predicted REEs were significantly correlated for all four equations (*p* < 0.001), with the Harris–Benedict equation demonstrating the strongest correlation in both men and women (r = 0.617, *p* < 0.001). Bland–Altman analysis revealed that the Harris–Benedict and Mifflin–St Jeor equations did not show a significant bias in the prediction of REE, whereas a significant overestimation error was shown for the FAO/WHO/UNU and Schofield equations.
Virkkunen (2009) [41]	Finland	Observational/case control	89 (49 habitually violent offenders; 40 HC)	Recidivistic offenders: [*n* = 17; 32.3 (10.7)]; non-recidivistic offenders: [*n* = 32; 32.9 (9.2)]; HCs: [*n* = 40; 33.7 (8.7)]	Habitually violent offenders who fulfilled the DSM-III criteria for antisocial personality disorder and alcohol dependence.	No intervention	Drug- and medication-free	Structured Clinical Interview for DSM-III-R, axes I and II	Gas exchange IC was used to determine glucose oxidation rates in the basal state and after 150 to 180 min of insulin clamp. Participants were in the fasted state and RER was used to infer substrate utilization. No mention in manuscript of sampling window used for IC measurement.	Offenders who committed at least one new violent crime during the 8-year follow-up had a mean NOG of 1.4 standard deviations lower than non-recidivistic offenders. In logistic regression analysis, NOG alone explained 27% of the variation in the recidivistic offending. The recidivistic offenders had higher baseline insulin levels than non-recidivistic offenders and healthy subjects, while IQ scores did not differ between recidivistic and non-recidivistic offenders (F1,44 = 0.93, *p* = 0.34).
Virkkunen (2007) [42]	Finland	Observational/case control	136 (67 P-APD; 29 F-APD; 40 HC)	P-APD = 33.2 (11.2); F-APD = 39.0 (8.1); HCs = 33.7 (8.7)	Male habitually violent offenders admitted from prison, fulfilling DSM-III-R criteria for persistent antisocial personality disorder (P-APD), in addition to previously incarcerated offenders (F-APD) and healthy male controls matched for age and weight	No intervention	All participants were drug free for at least 7 days prior to calorimetry	Structured Clinical Interview for DSM-III-R, axes I and II	Gas exchange IC was used to measure $\overset{\cdot }{V}$O_2_ and $\overset{\cdot }{V}$CO_2_ in the basal state and at 150 to 180 min of insulin clamp. Participants were in the fasted state and RER was used to infer substrate utilization. No mention in manuscript of sampling window used for IC measurement.	Habitually violent, incarcerated offenders with APD had significantly lower non-oxidative glucose metabolism, basal glucagon, and free fatty acids when compared with normal controls, but glucose oxidation and CSF 5-HIAA did not differ markedly between these groups. The effect sizes for lower non-oxidative glucose metabolism among incarcerated and non-incarcerated APD subjects were 0.73 and 0.51, respectively, when compared with controls, indicating that this finding was not explained by incarceration. Habitually violent offenders with APD have markedly lower glucagon and non-oxidative glucose metabolism when compared with healthy controls, and these findings were more strongly associated with habitual violent offending than low CSF 5-HIAA levels.

Abbreviations: BD-I = bipolar disorder type 1; HC = healthy control; BRMAS = Bech–Rafaelsen Mania Rating Scale; REE = resting energy expenditure; BMI = body-mass index; $\overset{\cdot }{V}$O_2_ = volume of inspired oxygen; $\overset{\cdot }{V}$CO_2_ = volume of expired carbon dioxide; DSM = Diagnostic and Statistical Manual of Mental Disorders; SMI = serious mental illness; DLW = doubly labelled water; FAO = Food and Agriculture Organization; WHO = World Health Organization; UNU = United Nations University; mg = milligram; TEE = total energy expenditure; kg = kilogram; kcal = kilocalorie; s = second; min = minute; d = day; SCZ = schizophrenia/schizophreniform; FFM = fat-free mass; LARN = Recommended Nutrients Assumption Levels; NOG = non-oxidative glucose metabolism; IC = indirect calorimetry; IQ = intelligence quotient; EE = energy expenditure; P-APD = persistent antisocial personality disorder; F-APD = former antisocial personality disorder; CSF = cerebrospinal fluid; 5-HIAA = 5-Hydroxyindoleacetic acid; m = metre; cm = centimetre; GAF = Global Assessment of Function;; kJ = kilojoules; HB = Harris–Benedict method; S = Schofield method; OUR = Owen’s method; HAMD = Hamilton Depression Rating Scale; PANSS = Positive and Negative States Scale; RCT = Randomized Controlled Trial; CBZ = Carbamazepine; TCA = Tricyclic Antidepressant; MAOI = Monoamine Oxidase Inhibitor; SAD = Seasonal Affective Disorder; SEM = Standard Error to the Mean; HOMA-IR = Homeostatic Model Assessment for Insulin Resistance.

## Data Availability

No new data were created or analyzed in this study. Data sharing is not applicable to this article.
